# Effect of melatonin, zinc sulfate, nano-melatonin, and nano-zinc oxide on mitochondria function and developmental competence of buffalo oocytes

**DOI:** 10.5455/javar.2024.k860

**Published:** 2024-12-29

**Authors:** Omaima Mohamed Kandil, Heba Elsaeed Elsehy, Nabil Mohamed Baker, Mohamed Asran Elbehiry, Sayed Ahmed Hattab

**Affiliations:** 1 Department of Animal Reproduction and Artificial Insemination, Veterinary Research Institute, Embryo and Genetic Resources Conservation Bank, National Research Centre, Cairo, Egypt; 2 Department of Theriogenology, Faculty of Veterinary Medicine, Damanhour University, Damanhour, Egypt; 3 Department of Infectious Diseases, Faculty of Veterinary Medicine, Damanhour University, Damanhour, Egypt; 4 Department of Theriogenology, Faculty of Veterinary Medicine, Alexandria University, Alexandria, Egypt

**Keywords:** Melatonin, zinc, nano-melatonin, nano-zinc, *In vitro* embryo development, mitochondria function, buffalo

## Abstract

**Objective:**

The aim of the current work is studying the effect of antioxidants and nano-­antioxidants on *in vitro* development and mitochondrial function of buffalo oocytes.

**Materials and Methods:**

Good and excellent Buffalo oocytes were *in vitro* matured: (1) tissue culture medium-199 (control group), (2) TCM-199 + melatonin (Mel) 10^−9 ^M (Mel group), (3) TCM-199 + zinc 10^−6^ M (Zn group), (4) TCM-199 + nano- Mel 10^−6 ^M (N-Mel group), and (5) TCM-199 + nano-zinc-oxide 10^−6 ^M (N-ZnO group) and incubated with CO_2_ 5% and 38.5°C for 22 hr. *In vitro*-matured oocytes were either stained for mitochondrial function or cultured for detection of embryo development.

**Results:**

The maturation rate of buffalo oocytes in the N-Mel and N-ZnO groups had a significant (*p* < 0.05) increase (91.89% and 93.64%, respectively) compared to the Mel group (85.78%) and Zn group (81.37%), and all groups were significantly higher than the control (73.16%). Mitochondrial intensity was significantly elevated (*p* < 0.05) in the N-Mel and N-ZnO groups than in oocytes matured in the Mel, Zn, or control groups. Rates of fertilization, cleavage, and transferable embryos of buffalo oocytes matured *in vitro* were significantly raised in the N-ZnO group (88.35%, 85.93%, and 30.71%, respectively) and the N-Mel group (86.74%, 82.75%, and 28.32%, respectively) (*p* < 0.05) when compared with the Mel group (82.46%, 77.25%, and 21.29%, respectively) and the Zn group (79.98%, 75.19%, and 19.68%, respectively), and all were increased significantly (*p *< 0.05) compared to the control group (71.76%, 68.7%, and 11.98%, respectively).

**Conclusion:**

Supplementation of maturation medium with Mel 10^−9^ M and zinc sulfate 10^−6^ M and nano-Mel 10^−6^ M and nano-zinc oxide 10^−6^ M improves buffalo oocyte maturation rates, mitochondrial function, and embryo development.

## Introduction

Buffalos are considered the main livestock wealth in most countries of the Mediterranean regions and South Asia. Nevertheless, its efficiency of reproduction has deteriorated due to a lack of animal selection and poor nutrition systems, so technologies of assisted reproduction (ART), including *in vitro* embryo production (IVEP), have been used to enhance reproductive efficiency in buffalo [1].

One of the challenges facing IVEP is oxidative stress (OS), which is considered the main factor that disturbs oocyte development and appears *in vitro* because of the inconsistency between both the producing and neutralizing of medium content of the reactive oxygen species (ROS) levels [2] formed due to cellular metabolism and enzyme reactions throughout the development of oocytes and other reasons associated with the composition of culture medium and conditions of *in vitro* culture [3].

One of the most important antioxidants used in maturation medium is melatonin (Mel), as it is a free radical scavenger, antioxidant, and anti-apoptotic agent [2] and enhances mitochondrial activity [4]. Mitochondrial distribution status in matured oocytes with Mel shows better maturation quality and even distribution of mitochondria in the ooplasm of porcine oocytes [5] leading to increased cytoplasmic and nuclear maturation rates in buffalo [6], cleavage, and transferable embryo rates of buffalo oocytes [2].

In addition, adequate zinc supply is necessary for the oocyte to make a fertilization-competent egg, and following egg fusion to the sperm, a rapid release of zinc, known as the zinc spark, enhances women’s egg activation [7] and increases the nuclear maturation rate of oocytes, cleavage, and transferable embryo rates in buffalo [8,9]. In addition, zinc improves the inner cell mass (ICM) numbers, which improves pregnancy rates after IVEP, as a low number of ICM is related to pregnancy failure in cattle [10].

As compared with antioxidants, nano antioxidants may show higher reactivity and solubility due to their extremely minuscule size [11] and greater antioxidant power [12]. The elevated concentration of nanostructured Mel significantly increases the oocyte maturation rates of buffalo [6] and increases the hatching rate and overproduces embryo cell number in bovine [13]. Zinc nanoparticles are used *in vitro,* as their addition significantly raises both maturation rates, cleavage rates, and the rate of transferable embryos of buffalo oocytes [9] and increases the bovine *in vitro* blastocyst rate [14].

Mitochondria have a great role during *in vitro* maturation of oocytes, so it is crucial for the oocyte to have the right number of functional mitochondria, as oocyte maturation needs a large adenosine 5-triphosphate (ATP) content required for progressive translation and transcription, so mitochondria showed a gradual increase in the copy number of mitochondrial DNA, and the distribution of mitochondria significantly changed [15].

However, according to our knowledge, there is little literature about the effect of Mel, zinc, nano-Mel, or nano-zinc on oocyte maturation, mitochondrial function, and oocyte development, especially in buffalo animals. Therefore, this study attempts to demonstrate the result of adding antioxidants (Mel and Zn) and nano antioxidants (N-Mel and N-ZnO) to the maturation medium on *in vitro* maturation, mitochondria function, and embryo development in buffalo.

## Material and Methods

### Ethical approval

Current work was performed in compliance with standard protocols unaccompanied by any annoyance or offense to the buffalo. Moreover, the steps of the experimental plan of action were accepted by the National Research Centre Ethics Committee, Cairo, Egypt (NRC, ID: 19/145).

Except as otherwise specified, all the chemical compounds used for the current study were obtained from Sigma-Aldrich, France. Systems of Particle Sizing, Inc., Santa Barbara, California, was used for the assessment of nanoparticles of zinc oxide (N-ZnO) size and zeta potential.

Chitosan (100–300 KD.) utilized in the current study was purchased from the company of Acros-Organic Thermo Fisher in New Jersey, United States (CAS: 9012-76-4, 90% deacetylation degree). Ethanol and acetic acid were obtained from El-Nasr Company for Intermediate Chemicals Industries, Cairo, Egypt.

*Nanoparticles of Mel-loaded chitosan (CMN) preparation:*

The preparation of chitosan nanoparticles was according to [6] dependent on the technique of ionic gelation, which depended on ions electrostatic interactions utilizing tripolyphosphate polyanion (TPP) particles for ion cross-connection with few modifications. Briefly, dissolve chitosan 1% in 1% acetic acid solution, then check pH to be 5.5, then filter by a filter of 0.22 μm. Mel was dissolved in chitosan solution at a concentration of 100 μmol/l, and one part of TPP solution (containing TPP 0.5 mg/ml) was adjusted to three parts of CS solution (1 mg/ml) for 60 min using magnetic stirring. Centrifugation of the prepared nanoparticles for 30 min at 10,000 rpm was then washed with special purified water that was ion-free. Washed nanoparticles were exposed to lyophilization and then stored at 4°C.

*Nanoparticles of Mel-loaded chitosan characterization:*

The constitution of particles of nano-Mel was evaluated utilizing a transmission electron microscope (HT7700; Hitachi, Japan). The Particle Sizing Systems, Inc., Santa Barbara, California, US, was used to examine the electrokinetic potential between the nanoparticles and the medium and measure the size of the Mel nanoparticles.

*Experiment I: Effect of Mel, zinc sulfate, nano-Mel, and nano-zinc oxide on in vitro maturation of oocytes of buffalo*

Obtaining ovaries of the buffalo animals were from El-Sharkawy slaughterhouse in Qalyubia Government in Egypt (2020–2023) and transported in a small tank with normal saline solution 0.9% (NSS, NaCl). Ovaries were terminated, then washed in pre-heated NSS at 37°C many times and maintained in this warm NSS till aspiration time. Cumulus-oocyte complexes were taken from follicles with a diameter of 2–8 mm by a needle with an 18-gauge attached to a disposable sterile syringe (10 ml) containing 1 ml of aspiration medium, which was composed of phosphate-buffered saline (PBS) + bovine serum albumin (BSA) 6 mg/ml + gentamicin 50 μg/ml. Following follicle aspiration, follicular fluid was put into a Falcon tube and left in a 37°C water bath to give it a chance to settle down for about 15 min. Aspirated buffalo oocytes were evaluated utilizing a 90x magnification stereo microscope and then washed using the aspiration medium three times [9].

Oocyte’s quality was classified into four groups based on cumulus layers and cytoplasm homogenization (excellent, good, fair, and denuded) according to [2].

*Excellent:* Oocytes surrounded with more than five layers of completely expanded cumulus cells and uniformly granulated cytoplasm.

*Good:* Cumulus cells arranged from three to five layers and dark, evenly granulated ooplasm.

*Fair:* Aspirated oocytes are partly surrounded by cumulus cells, and their cytoplasm has little granulation.

*Denuded:* No cumulus cells encircled oocytes and covered by zona pellucida only with no cumulus cells.

Oocytes with excellent and good quality (Fig. 5A) (*n = *1805) were *in vitro* matured in five groups. (1) TCM-199 as a control group (*n = *434), (2) TCM-199 + Mel 10^−9^ M (*n = *363), (3) TCM-199 + zinc sulfate 10^−6^ M (*n = *389), (4) TCM-199 + Nano-Mel 10^−6^ M (*n = *318), and (5) TCM-199 + nano-zinc oxide 10^−6^ M (*n = *301) with the addition of fetal calf serum (FCS) 10% + 50 μg/ml gentamicin + 10 μg/ml follicular stimulating hormone (FSH). Buffalo oocytes were matured *in vitro* for 22 h in a humidified CO_2_ incubator with 5% CO_2_ at 38.5°C.

Cytoplasmic maturation was examined depending on the extent of growth and development of cumulus cells and categorized into four grades [2]. G0: Oocytes are without any expansion of cumulus cells.

GI: Oocytes have slight cumulus expansion.

GII: Oocytes have moderate cumulus expansion.

GIII: Oocytes are with complete (full) expansion of cumulus cells.

Nuclear maturation of buffalo oocytes that matured *in vitro* was determined according to the existence of the first polar body (Pb) in the oocyte after cumulus cells surrounding oocytes were removed by gentle pipetting. The presence of the first Pb was observed using a 200X magnification inverted microscope. Oocytes showing the first Pb are considered matured oocytes (MII). This experiment was replicated six times for all groups.

*Experiment II: Effect of Mel, zinc sulfate, nano-Mel, and nano-zinc oxide on mitochondrial distribution and intensity of buffalo oocytes matured in vitro*

Buffalo oocytes, which previously *In vitro* matured (*n = *150) from the five groups were fixed using paraformaldehyde 2% to maintain the spherical shape of oocytes, then washed with PBS supplemented with polyvinyl-pyrrolidone (0.1%) and then PBS containing Triton (0.2%) to increase cell membrane permeability to stain. *In vitro* matured oocytes of buffalo were stained by a fluorescent probe specific for mitochondria, Mito Tracker Red FM stain (Thermo Fisher). Stained oocytes were examined for mitochondrial distribution utilizing a confocal microscope (Zeiss LSM 710) depending on their morphology. *In vitro* matured oocytes were kept in phosphate-buffered saline with Mito Tracker Red FM 500 nM concentration for about 30 min in the incubator at a temperature of 37°C. Stained oocytes of buffalo were washed in PBS twice and then added into PBS supplemented with 5 µg/ml DAPI and incubated together. DAPI stain is used to detect the first Pb and counterstain the nucleus in matured buffalo oocytes. Examination of DAPI stain was done at 20X magnification of a confocal microscope in emission 4601 mm and excitation 358 mm wavelengths. Stained buffalo oocytes were subsequently washed in phosphate-buffered saline and then were visualized in a 12 mm diameter culture plate with a glass bottom (Thermo Fisher) by the confocal microscope. The fluorescence of Mito Tracker red stain was determined by an argon laser in a 644 nm emission filter and 581 nm excitation line. We examined a cross section of each stained oocyte in the nucleus visibility plane. The evaluation of the mitochondrial distribution was as follows: (1) Peripheral distribution of mitochondria was in oocytes, in which only mitochondria were located in the inner surface of the zona pellucida; (2) semi-peripheral mitochondrial distribution in which mitochondria were arranged toward the zona pellucida inner surface but distribution in the inner region was inhomogeneous; (3) semi-diffused distribution in which mitochondria were found in the center and inhomogeneous distribution toward the oocyte wall; (4) diffused mitochondrial distribution was in oocytes with homogenous mitochondrial distribution in the whole inner region of the cytoplasm. The intensity of mitochondria was examined using the confocal microscope software [16]. This experiment was replicated for all groups of *in vitro* matured buffalo oocytes three times, with each replicate (*n = *10 oocytes).

*Experiment III: Effect of Mel, zinc sulfate, nano-Mel, and nano-zinc oxide on in vitro embryo development*

Following the *in vitro* maturation step of buffalo oocytes, according to [9], matured oocytes of five groups, the control group (*n = *304), the Mel group (*n = *281), the Zn group (*n = *296), the N-Mel group (*n = *270), and the N-ZnO group (*n = *256) were washed in Fert-TALP medium (fertilization Tyrode’s albumin lactate pyruvate medium) with 1 µg/ml heparin + 6 mg/ml BSA, which is free from fatty acid, + 2.5 µg/ml hypotaurine + 5 µg/ml gentamicin. The straw of frozen semen was thawed in a warm water bath at a temperature of 37°C then washed with 3 ml Sperm-TALP medium which was previously added to 1 μg/ml heparin and 3 mg/ml BSA + 2.5 µg /ml hypotaurine + 50 µg/ml gentamicin, and centrifugated at ١٨٠٠ rpm for ١٠ min. After discarding the supernatant, the semen pellet that remained was remixed with ٣ ml of Fert-TALP medium and centrifuged once more at ١٨٠٠ rpm for ٥ min. After completion of the two steps of centrifugation, the supernatant was discarded, and the remaining semen pellet was added to Fert-TALP. Before being placed in a four-well plate, the sperm concentration of prepared semen was modified to 1 × 10^6^ spermatozoa/ml. Both semen and oocytes were incubated in a 5% CO_2_ humidified atmosphere at 38.5°C for 18 h. Assessment of fertilization by the presence of second Pb. *In vitro* fertilized oocytes of buffalo were kept in culture medium, which was modified synthetic oviductal fluid (mSOF) medium added to 50 µg/ml gentamicin + 5 mg/ml BSA, and were cultured and kept at 38.5°C in a 5% CO_2_ incubator for 7 days. Monitoring of the cleavage of oocytes was on days 2, 4, and 7. Every 2 days, the culture medium had to be changed. According to [2], the fertilization, cleavage, and transferable embryo rates (morula and blastocyst) were detected. In this experiment, each group had six replicates.

### Statistical analysis

Data from the current investigation were displayed using mean ± standard error (SE). Assessment of significant differences was assessed using analysis of variance or the *Chi*-Square Test followed by a *Post Hoc* test. The statistical analysis was done by SPSS of Windows, Version 28.0, SPSS Inc., Chicago, IL.

## Results

### Morphology of prepared capsulated Mel nanoparticles (CMNs) and their characterization

**Figure 1.**
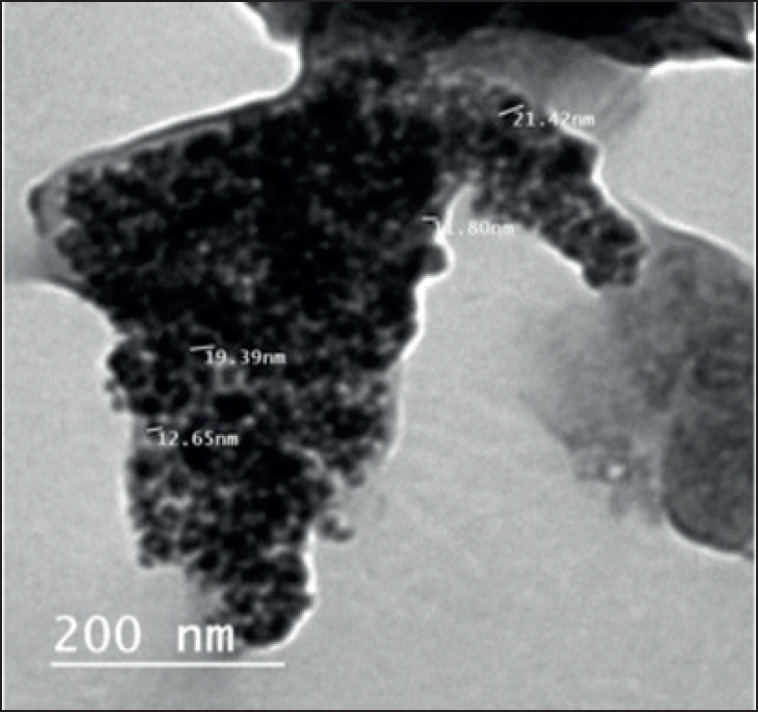
Transmission electron microscope image of CMN with scale 200 nm at 25°C.

Transmission electron microscopy was used for examination of the morphology of prepared CMN samples (Fig. 1), which demonstrates that the shape of prepared particles is semi-spherical with an 11–22 nm size distribution and shows uniform distribution of the particles in every part of the sample of Mel-loaded chitosan nanoparticles. The mean particle size was determined by noticing both maximum and minimum diameters for most particles, which revealed an average particle size of up to 30 nm. The effectiveness of encapsulation was determined at 34%, and drug loading of the produced N-Mel was measured at 16%.

**Figure 2.**
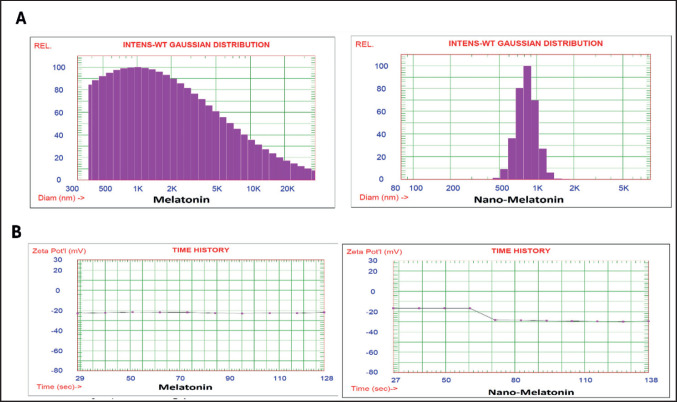
A) Analysis of intensity-weighted gaussian distribution (solid particle) of melatonin and nano-melatonin particles B) Average zeta potential of melatonin and nano-melatonin particles

Mel and Mel nanoparticles characterization (Fig. 2A) showed the analysis of distributions of intensity-weighted Gaussian (means ± SD) of Mel nanoparticles was 842.2 nm with 0.036 variances; however, it was 3738.9 nm in Mel with variance 2.686. The average Zeta potential (the dispersion stability) was −29.49 mV in Mel nanoparticles and −21.94 mV in Mel (Fig. 2B).

### Characterization of Zn sulfate and nano-zinc oxide (N-ZnO)

**Figure 3.**
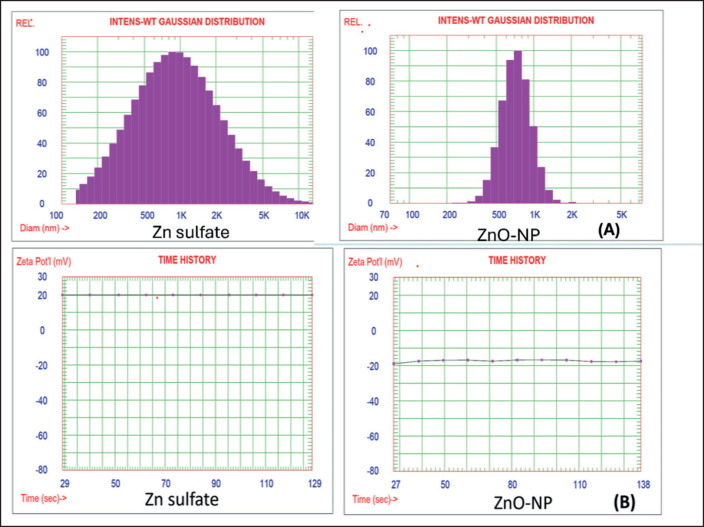
A) Analysis of intensity of weighted gaussian distribution (solid particle) of ZnO-NP particles and Zn sulfate, B) Average zeta potential of ZnO-NP particles and Zn sulfate

Analysis of distributions of the intensity-weighted Gaussian (means ± SD) in nanoparticles of zinc oxide (N-ZnO) was 744.4 nm with variances of 0.076; however, it was 1316.3 nm in zinc sulfate with a 0.771 variance (Fig. 3A). In addition, the dispersion stability (average zeta potential) in nano-zinc oxide (N-ZnO) was −17.47 mV, and in zinc sulfate, it was 19.78 mV (Fig. 3B).

### Experiment I: Effect of Mel, zinc sulfate, nano-Mel, and nano-zinc oxide on in vitro maturation rates of buffalo oocytes

*Cytoplasmic maturation rate (cumulus expansion)*

This study showed that the addition of Mel 10^−9^ M, zinc sulfate 10^−6^ M, nano-Mel 10^−6^ M, and nano-Zn-O 10^−6^ M to the maturation medium of buffalo oocytes significantly (*p* < 0.05) increased the percentage of oocytes with Grade III cumulus cell expansion (GIII) (Table 1; Fig. 5B) (58.52% ± 0.96%, 52.32% ± 0.85%, 66.12% ± 0.95%, 67.64% ± 1.17%, respectively) when compared to control (43.3% ± 1.29%). In addition, Gll was significantly (*p *< 0.05) greater in Mel 10^−9^ M, zinc sulfate 10^−6^ M, nano-Mel 10^−6^ M, and nano-Zn-O 10^−6^ M (24.41% ± 0.71%, 24.67% ± 1.04%, 28.57% ± 0.68%, and 27.65% ± 1.21%, respectively) when compared with TCM-199 (16.24% ± 0.72%). On the other hand, Gl cumulus expansion of oocytes matured with Mel, Zn sulfate, nano-Mel, and nano-Zn-O was significantly lower (*p *< 0.05) (9.92% ± 0.51%, 13.12% ± 0.84%, 3.19% ± 0.39%, and 2.22% ± 0.48%, respectively) than the control group (17.68% ± 0.77%). Grade 0 was significantly (*p *< 0.05) lower in Mel, Zn sulfate, nano-Mel, and nano-Zn-O (7.17% ± 1.01%, 9.89% ± 1.09%, 2.12% ± 0.58%, and 2.48% ± 0.42%, respectively) than the control group (22.77% ± 0.92%). There is a significant (*p* < 0.05) increase in Glll and Gll cumulus cell expansion of oocytes supplemented with nano-Mel and nano-Zn-O as compared with oocytes supplemented with Mel and Zn sulfate. Gl and G0 had a significant (*p *< 0.05) decrease in oocytes supplemented with nano-Mel and nano-Zn-O as compared with oocytes supplemented with Mel and Zn sulfate.

*Nuclear maturation rate*

Mature oocytes were examined for the presence of first Pb as an indication for meiosis II and found that the maturation rate in groups supplemented with Mel, Zn sulfate, nano-Mel, and nano-Zn-O was (85.78% ± 1.07%, 81.37% ± 1.36%, 91.89% ± 0.77%, and 93.64% ± 0.87%, respectively) and was significantly (*p* < 0.05) higher than the control (73.16 ± 0.67%). In addition, nano-Mel and nano-Zn-O groups had a significant (*p* < 0.05) higher maturation rate than Mel and Zn sulfate groups, and the Mel group had a significant increase in maturation rate than the Zn sulfate group (Table 2; Fig. 5C).

### Experiment II: Effect of Mel, zinc sulfate, nano-Mel, and nano-zinc oxide on mitochondrial intensity and mitochondrial distribution of in vitro matured buffalo oocytes

Mitochondrial intensity was significantly (*p < *0.05) higher in Nano-Mel (423.21 ± 47.47) and nano-zinc oxide (413.44 ± 37.92) when compared with Mel (289.69 ± 14.52), zinc sulfate (277.99 ± 34.49), and the control group (205.02 ± 25.36) (Table 3).

**Table 1.** Effect of melatonin, zinc sulfate, nano-melatonin and nano zinc oxide on cumulus expansion of buffalo oocytes.

Item	Total oocyte	Glll		Gll		G1		G0	
		No.	%	No.	%	No.	%	No.	%
Control	434	185	43.3 ± 1.29^d^	72	16.24 ± 0.72^c^	76	17.68 ± 0.77^a^	101	22.77 ± 0.92^a^
Melatonin 10^−9^ M	363	211	58.52 ± 0.96^b^	90	24.41 ± 0.71^b^	37	9.92 ± 0.51^c^	25	7.17 ± 1.01^c^
Zinc sulfate 10^−6^ M	389	206	52.32 ± 0.85^c^	96	24.67 ± 1.04^b^	49	13.12 ± 0.84^b^	38	9.89 ± 1.09^b^
Nano-melatonin (10^−6^ M)	318	211	66.12 ± 0.95^a^	91	28.57 ± 0.68^a^	10	3.19 ± 0.39^d^	6	2.12 ± 0.58^d^
Nano zinc oxide (10^−6^ M)	301	203	67.64 ± 1.17^a^	85	27.65 ± 1.21^a^	6	2.22 ± 0.48^d^	7	2.48 ± 0.42^d^

Replicates = 6, Means within the same column carry different superscripts (a, b, c and d) are significantly different at *p *< 0.05, M: mole.

**Table 2.** Effect of melatonin, zinc sulfate, nano-melatonin and nano zinc oxide on nuclear maturation rate of buffalo oocytes.

Item	Total oocytes	Oocytes number with first Pb	Nuclear maturation rate %	Oocytes number without first Pb	Non matured oocytes %
Control	434	319	73.16 ± 0.67^d^	115	26.84 ± 0.67^a^
Melatonin 10^−9^ M	363	312	85.78 ± 1.07^b^	51	14.22 ± 1.07^c^
Zinc sulfate 10^−6^ M	389	313	81.37 ± 1.36^c^	76	18.63 ± 1.36^b^
Nano-melatonin (10^−6^ M)	318	293	91.89 ± 0.77^a^	25	8.11 ± 0.77^d^
Nano Zn-O (10^−6^ M)	301	282	93.64 ± 0.87^a^	19	6.36 ± 0.87^d^

Replicates = 6, Means within the same column carry different superscripts (a, b, c and d) are significantly different at *p *< 0.05, M:mole, total oocytes: number of good and excellent oocytes.

**Figure 4.**
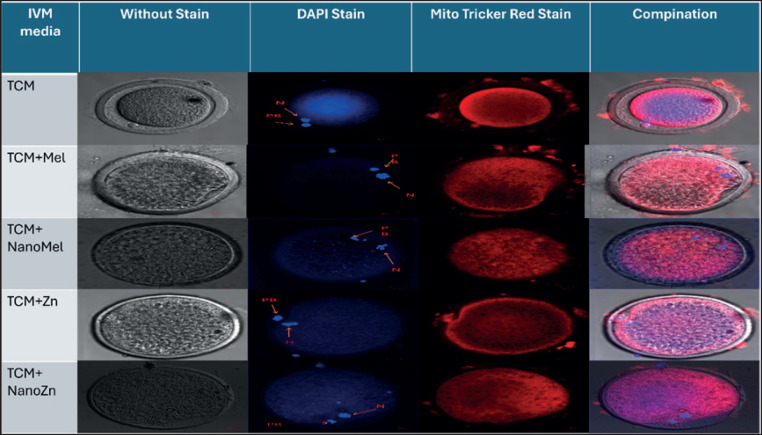
Detection of viability and mitochondrial function on in-vitro matured buffalo oocytes using a confocal microscope (Ziess LSM 710) , matures oocytes without staining, matured oocytes stain with DAPI stain, *N = *nucleus, Pb = polar body, TCM media show,Simi-peripheral distribution of mitochondria, TCM-199 + melatonin 10^−9 ^M media showed Simi-diffused distribution of mitochondria, TCM-199 +Zn sulfate 10^−6^ M media showed Simi-peripheral distribution of mitochondria, TCM-199 + nano-melatonin 10^−6 ^M media and TCM-199 +Zn Oxid nanoparticle 10^−6 ^M media showed a Diffused distribution of mitochondria of *in vitro* matured oocytes stained with Mito Tricker stain, Combination of three pictures.

Peripheral distribution of mitochondria was significantly (*p *< 0.05) higher in the control group (80% ± 5.77%) than in other groups that had no peripheral distribution (0%) and had a highly significant percentage of semi-peripheral mitochondria distribution (20% ± 5.77%) than in other groups. On the other hand, the addition of nano-Mel and nano-Zn gives significantly (*p* < 0.05) higher diffused distribution (80% ± 5.77 and 90% ± 5.77%, respectively) than Mel and zinc groups (60% ± 11.55% and 20% ± 10.0%, respectively) and is not found in the control group (0%). Semi-diffused mitochondrial distribution of the zinc sulfate group was significantly (*p* < 0.05) elevated (70% ± 10.0%) than Mel, nano-Mel, and nano-zinc (30% ± 10.0, 10% ± 5.77, and 10% ± 5.77%, respectively) but not found in the control group (0%) (Table 4; Fig. 4).

### Experiment III: Effect of Mel, zinc sulfate, nano-Mel, and nano-zinc oxide on buffalo oocyte development

**Figure 5.**
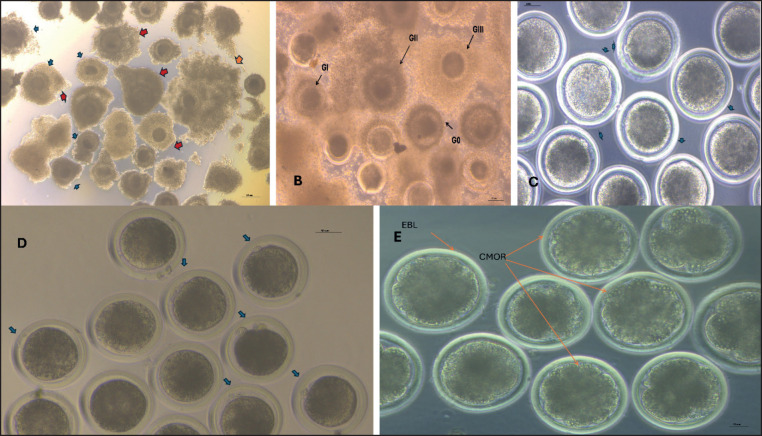
Buffalo oocyte quality and developmental competence, A) Buffalo oocyte quality, Red arrow = Excellent oocytes and Black arrow = Good oocytes, B) Cumulus expansion in matured buffalo oocytes GIII = full expansion and GII=Modred expansion, GI = few expansions and G0=no expansion, C) *In vitro* matured buffalo oocytes with arrow showed first Pb) matured in nano-melatonin (10^−6 ^) media, D) *In vitro* fertilized buffalo oocytes with arrow showed second polar body (second Pb),E) *In vitro* produced buffalo’s transferable embryo of Zn oxide nano-particles, CMOR = compact morula, EBL = early blastocyst.

Fertilization rate was significantly (*p < *0.05) greater in Nano-Mel and Nano Zn (86.74% ± 0.93% and 88.35% ± 1.39%, respectively) when compared to Mel, Zn, and control (82.46% ± 1.24%, 79.98% ± 1.4%, and 71.76% ± 0.95%, respectively) (Fig. 5D). In addition, Mel and zinc groups have a significant increase (*p* < 0.05) in fertilization rate compared to the control group. Cleavage rate and transferable embryo rate were significantly (*p* < 0.05) higher in Nano-Mel (82.75% ± 0.73% and 28.32% ± 0.69%, respectively) and nano-Zn (85.93% ± 1.4% and 30.71% ± 1.31%, respectively) when compared with Mel (82.46% ± 1.24% and 77.25% ± 1.44%, respectively), Zn (79.98% ± 1.4% and 75.19% ± 0.81%, respectively), and all were significantly (*p *< 0.05) higher than the control group (68.7% ± 0.99% and 11.98% ± 0.68%, respectively) (Table 5; Fig. 5E).

**Table 3.** Effect of melatonin, zinc sulfate, nano-melatonin and nano zinc oxide on mitochondrial Intensity of buffalo oocytes.

Item	Number of imaged oocytes	mitochondrial intensity mean ± SE
Control	30	205.02 ± 25.36^ b^
Melatonin 10^−9^ M	30	289.69 ± 14.52^ b^
Zinc sulfate10^−6^ M	30	277.99 ± 34.49^ b^
Nano-melatonin(10^−6^ M)	30	423.21 ± 47.47^ a^
Nano zinc oxide (10^−6^ M)	30	413.44 ± 37.92^ a^

Means within the same column carry different superscripts (a, b) are significantly different at *p* < 0.05 Replicates = 3, M: mole.

## Discussion

Our study revealed that nano-Mel and nano-zinc oxide improve nuclear maturation and cytoplasmic expansion rates of buffalo oocytes as compared to Mel, zinc, and control groups. These results are in agreement with [6], whose work revealed a significant increase in cytoplasmic (GIII %) and nuclear maturation rates of oocytes of buffalo matured with Mel-loaded chitosan nanoparticles (51.57% and 94.04%, respectively) than those matured with Mel (47.5% and 88.7%, respectively) and the control group (44% and 79.67%, respectively). In addition, the results of the nano-zinc oxide group agreed with [9], whose results demonstrated that the nano-zinc group had a higher percentage of Glll cytoplasmic maturation (59.75%) with a significant difference (*p < *0.05) than the zinc and control groups (52.93% and 36.80%, respectively) and a significantly higher nuclear maturation rate (92.43%) than the zinc group (86.98%) and control group (80.11%). Nano-Mel protects mice oocytes from ROS during *in vitro* maturation procedures, upregulating the antioxidant genes and downregulating the pro-apoptotic (BAX and CASP3) genes in buffalo oocytes [6]. Jahanbin et al. [14] Clarify the positive effect of nano-Zn-O, which increases the activity of superoxide dismutase (SOD) in cumulus cells and decreases apoptosis and DNA damage of bovine oocytes. In addition, total GSH concentrations in oocytes and cumulus cells significantly increased when nano-Zn was used in the maturation medium of bovine oocytes [17]. Nano antioxidants have potent radical scavenging and quenching capacities that have shown greater antioxidant power and more resistance to microenvironments than natural antioxidants [12].

**Table 4.** Effect of melatonin, zinc sulfate, nano-melatonin and nano zinc oxide on mitochondrial distribution of buffalo oocytes.

Mitochondria distribution	Matured oocytes	Diffused		Semi-diffused		Semi-peripheral			Peripheral	
		No.	%	No.	%		No.	%	No.	%
Control	30	0	0^c^	0	0^c^		6	20 ± 5.77^a^	24	80 ± 5.77^a^
Mel 10^−9^ M	30	18	60 ± 11.55^b^	9	30 ± 10.0^b^		3	10 ± 5.77^ab^	0	0^b^
Zn sulfate10^−6^ M	30	6	20 ± 10.0^c^	21	70 ± 10.0^a^		3	10 ± 5.77^ ab^	0	0^ b^
Mel-NPs 10^−6^ M	30	24	80 ± 5.77^ab^	3	10 ± 5.77^bc^		3	10 ± 0^ ab^	0	0^ b^
ZnO-NPs10^−6^ M	30	27	90 ± 5.77^a^	13	10 ± 5.77^bc^		0	0^b^	0	0^ b^

Means within the same column carry different superscripts (a, b, c) are significantly different (*p* < 0.05) Replicates = 3, M: mole.

**Table 5.** Effect of melatonin, zinc sulfate, nano-melatonin and nano zinc oxide on buffalo oocytes development.

Item	Total oocytes*	Fertilization rate		Two cell		Four cell		Cleavage		Transferable embryo rate	
		No.	%	No.	%	No.	%	No.	%	No.	%
Control	304	218	71.76 ± 0.95^c^	48	31.96 ± 1.4^a^	84	56.07 ± 1.35^a^	150	68.7 ± 0.99^c^	18	11.98 ± 0.68^c^
Melatonin 10^−9^ M	281	232	82.46 ± 1.24^b^	52	29.17 ± 0.42^a^	89	49.54 ± 1.22^b^	179	77.25 ± 1.44^b^	38	21.29 ± 1.18^b^
Zinc 10^−6^ M	296	237	79.98 ± 1.4^b^	55	30.66 ± 2.49^a^	88	49.66 ± 3.11^b^	178	75.19 ± 0.81^b^	35	19.68 ± 1.87^b^
Nano-melatonin 10^−6^ M	270	234	86.74 ± 0.93^a^	56	28.77 ± 0.95^a^	83	42.9 ± 1.41^c^	194	82.75 ± 0.73^a^	55	28.32 ± 0.69^a^
Nano zinc 10^−6^ M	256	226	88.35 ± 1.39^a^	44	22.97 ± 1.45^b^	90	46.32 ± 0.8^bc^	194	85.93 ± 1.4^a^	60	30.71 ± 1.31^a^

Means within the same column carry different superscripts (a, b, c) are significantly different at* p* < 0.05 replicates = 6, *Oocytes with first and second polar bodies, TER: transferable embryo rate, M: mole.

This study revealed that the Mel group had significantly higher maturation rates when compared with control groups, which comes in the same line with [18], whose results showed that the nuclear maturation rate of buffalo oocytes in the Mel group (85.20%) was higher than the control group (69.20%) with a significant difference, and [6], who demonstrated that Mel had significantly elevated both cytoplasmic and nuclear maturation rates in buffalo than the control group, and the same effect on bovine oocytes [19,20]. These positive effects of Mel are due to improved organelle normal distribution and increased intracellular glutathione (GSH) and SOD expression levels [19]. GSH is crucial for cumulus expansion *in vitro* procedures [21]. In addition, Mel significantly up-regulated the expressions of genes related to decreasing OS (BCL2, GDF9, and BMP15) and down-regulated the BAX gene related to apoptosis [6]. In contrast, Mel during *in vitro* maturation did not affect the maturation rate of *in vitro* matured bovine oocytes [22], but this disagreement may be because of the different concentrations of Mel used (10^−6^ M) as compared to our work (10^−9^ M).

The current study demonstrated that both maturation rates (cytoplasmic and nuclear) of the zinc group were significantly higher than those of the control group (*p* < 0.05). This is in agreement with [9], who revealed that zinc elevated Glll and Gll cumulus expansion rates and nuclear maturation rate of buffalo oocytes significantly higher than the control group (*p* < 0.05) and [23], who demonstrated that the proportion of matured buffalo oocytes was significantly elevated in the zinc-supplemented group in maturation medium compared to the control group (*p *< 0.05). Xiong et al. [24] revealed that zinc improved yak Cumulus expansion and nuclear maturation by elevating intracellular GSH and SOD activity in addition to decreasing ROS levels. Zinc decreased apoptosis as a result of the up-regulation of anti-apoptotic genes and the down-regulation of pro-apoptotic genes [23]. However, [25] proved that zinc 1.5 µg/ml did not modify cumulus expansion, but this can be explained by the adverse effect of zinc when used with high concentration (50–200 µM), as with increased zinc concentration, the maturation rate is lower than in the control group [26].

Results of mitochondrial intensity revealed that the nano-Mel and nano-zinc oxide groups had higher intensity of mitochondria than the Mel, zinc, and control groups with a significant difference (*p* < 0.05) and also had the highest percent of mitochondrial diffusion among groups. Because of nanoparticles’ characteristics, they increase the half-life of bioactive antioxidant components loaded on them and improve their positive effects during *in vitro* procedures, as nanoparticles prevent the deterioration of antioxidants. These active carriers are able to regulate the freeing of carried biomolecules, such as natural antioxidants, and carry chemicals to the target tissues using lower doses [27]. In addition, Mel enhances mitochondrial distribution to 60% diffused in current work, which comes in agreement with [18] in buffalo and [5] in porcine, as Mel enhances mitochondrial activity and increases the concentration of ATP [4]. There is little literature about mitochondrial function, according to our knowledge, using antioxidants and nano-antioxidants, especially in buffalo. All were mentioned about other species such as porcine, mice, and human.

According to our results, the nano-Mel and nano zinc-oxide groups have higher fertilization, cleavage, and transferable embryo rates than the Mel and zinc groups with significant differences (*p *< 0.05), and all were significantly higher than the control group. Results of nano-Mel come in agreement with [28] whose results revealed that Mel nanoparticles improved the quality of the mice oocytes as it increased embryo development and the blastocyst production rate using a lower dosage than Mel and [13] whose results showed that Mel-loaded lipid-core nano-capsules 10^−9^ M had a significantly higher hatching rate 92% compared to Mel group 61% but cleavage rate not affected, and nano-Mel protects oocytes against cell apoptosis and OS during bovine *in vitro* embryo culture as it decreased cell apoptosis and levels of ROS, in contrast, nano-Mel increased embryo cell number. Kandil et al. [9] revealed that buffalo oocytes matured with nano-zinc had significantly higher cleavage rates and transferable embryo rates than zinc, and both are significantly higher than the control group and [14] in bovine whose results clarify the positive effect of nano-Zn-O, which increases SOD activity, decreases apoptosis and DNA damage, and improves embryo development rates. In addition, total GSH concentrations significantly increased when nano-Zn was used in the maturation medium of bovine oocytes [17]. However, another study found that oocyte exposure to Zn-O nanoparticles at 12.5 μg/ml inhibits early embryonic development in fertilized chicken eggs, so they are considered embryo-toxic [29]. As carriers, there is an additional advantage of nanoparticles: they are able to make hydrophobic compounds with increased solubility and improve the dissolving of the active ingredients [30]. Nanotechnology has a crucial role in IVEP as it found the exit for most impairments facing embryo development since it provides nano-materials of antioxidants and hormones that are easier to deliver *in vitro* inside gametes and embryos, enhancing their ability for developmental competence [31].

Mel gives significantly (*p* < 0.05) higher fertilization, cleavage, and transferable embryo rates than the control group, which comes in agreement with [2] and [18], whose results revealed that Mel significantly increases cleavage rate and percentage of transferable embryos of *in vitro* matured buffalo oocytes as well as in bovine [19]. Mel increased concentrations of GSH during *in vitro* maturation and had many benefits for embryos: they improved their capability for successive development and increased the percentage of embryos that came to the blastocyst stage [21]. However, [22] proved that Mel with a concentration of 10^−6^ M during *in vitro* maturation did not affect embryo development in bovine. In addition, zinc gives better embryo development than the control group, according to our results, which is consistent with the results of [9], as zinc supplementation decreases ER stress by providing essential metal ion transporters for the maturation of oocytes and subsequent embryonic development in porcine [8]. Zn also improved ICM cell number, leading to improved pregnancy retention rates after *in vitro* culture embryo transfer [10]. In contrast, [25] proved that zinc 1.5 µg/ml did not affect the cleavage rate of bovine oocytes.

The current study revealed the relation between buffalo oocyte maturation rates, mitochondrial intensity and distribution, and embryo development. Results in relation to mitochondrial function indicate that significant, highly mature rates are obtained from oocytes with higher mitochondrial intensity and characterized by diffused mitochondrial distribution in the ooplasm, as in groups of nano-Mel and nano-zinc oxide. For example, the highest maturation rates obtained with zinc oxide nanoparticles and nano-Mel were 93.64% ± 0.87% and 91.89% ± 0.77%, respectively, and significantly (*p* < 0.05) higher mitochondrial intensity (413.44 and 423.21, respectively) with mitochondrial distribution were 90% ± 5.77% and 80% ± 5.77%, respectively. The diffused distribution gives the highest fertilization, cleavage, and transferable embryo rates. In contrast, the lowest maturation rate was for the control, 73.16 ± 0.67 with a mitochondrial distribution of 80% peripheral and 20% semi-peripheral, and no diffused or semi-diffused were found.

## Conclusion

The addition of antioxidants (Mel and zinc sulfate) and nano antioxidants (nano-Mel and nano-zinc oxide) improves maturation rates, mitochondrial function, and developmental competence of buffalo oocytes. In addition, nano antioxidants give significantly higher maturation rates, mitochondrial function, and embryo development of buffalo oocytes than antioxidants.

## List of abbreviations

ART, Assisted reproductive technologies; BSA, Bovine serum albumin; CMN, Capsulated melatonin nanoparticle; IVEP, In-vitro embryo production; MEL, Melatonin; MSOF, Modified synthetic oviductal fluid; N-Mel, Nano-melatonin; N-ZnO, Nano-zinc-oxide; OS, Oxidative stress; TCM, Tissue culture medium; TPP, Tripolyphosphate polyanion; ROS, ROS; Zn, Zinc.
